# Non-Contact Universal Sample Presentation for Room Temperature Macromolecular Crystallography Using Acoustic Levitation

**DOI:** 10.1038/s41598-019-48612-4

**Published:** 2019-08-27

**Authors:** R. H. Morris, E. R. Dye, D. Axford, M. I. Newton, J. H. Beale, P. T. Docker

**Affiliations:** 1 School of Science and Technology, Nottingham Trent University, Nottingham, NG11 8NS UK; 2 Diamond Light Source, Harwell Science and Innovation Campus, Oxfordshire, OX11 0DE UK

**Keywords:** Characterization and analytical techniques, Acoustics

## Abstract

Macromolecular Crystallography is a powerful and valuable technique to assess protein structures. Samples are commonly cryogenically cooled to minimise radiation damage effects from the X-ray beam, but low temperatures hinder normal protein functions and this procedure can introduce structural artefacts. Previous experiments utilising acoustic levitation for beamline science have focused on Langevin horns which deliver significant power to the confined droplet and are complex to set up accurately. In this work, the low power, portable TinyLev acoustic levitation system is used in combination with an approach to dispense and contain droplets, free of physical sample support to aid protein crystallography experiments. This method facilitates efficient X-ray data acquisition in ambient conditions compatible with dynamic studies. Levitated samples remain free of interference from fixed sample mounts, receive negligible heating, do not suffer significant evaporation and since the system occupies a small volume, can be readily installed at other light sources.

## Introduction

Efficient micro-dimensional sample delivery is becoming increasingly important to Macromolecular Crystallography (MX) at synchrotron light sources. Improvements in X-ray optics now allow for sub-micron beam profiles, increasing the need for the development of novel methods in sample delivery and alignment. Currently, by far the most common strategy, which accounted for 97% of the published X-ray structures in 2017, relies upon a cryo-cooled sample. Cryo-cooling is principally used to reduce sample damage from the effects of the ionizing X-ray beam whilst measurements are made. However, cryogenic temperatures are not the natural state of biological molecules and the cryo-cooling process can be terminally detrimental to the crystal architecture^1^. Cryo-cooling also prohibits the observation of biological reactions in real-time and potentially locks the protein in an unrepresentative conformation^2^. In this respect, effective data acquisition methods for room temperature crystallography represent a valuable tool for structural biologists^3^, albeit operating within the limits of protein crystal packing and order.

The development of X-ray Free Electron Lasers (XFELs) has led to the evolution of novel sample delivery strategies, which are now also being applied to synchrotron light sources. The brilliance of the XFEL pulse allows for a single, still diffraction image to be collected before the protein crystal is destroyed, removing the need for sample cryo-cooling. Therefore, sample delivery systems have been developed which channel large quantities of protein crystals into the XFEL beam at room temperature. These methods, including dynamic virtual nozzles^4^, lipidic cubic phase (LCP) extruders^5^, acoustic droplet ejectors (ADE)^6^, concentric-flow electrokinetic injectors^7^ and conveyor belts^8^, all share a more dynamic approach to sample delivery. Since these systems operate at room temperature, samples are much closer to the typical operating temperatures of functional proteins, bringing the possibility for small molecule diffusion during the X-ray data collection. Room temperature experiments therefore allow for reaction dynamics to be probed and for structure artefacts present in cryo-cooled samples to be avoided.

In recent years synchrotron MX beamlines have adopted similar methods to those created for XFELs as some are directly transferable, such as the fixed-targets^9^ and LCP extruders^10^. The success of this transfer has even inspired dedicated serial MX beamlines at PETRA^11^, Germany, at MAX IV^12^, Sweden, and also at ESRF, France^13^, allowing for novel sample delivery and alignment methods to be explored, although some of these techniques introduce physical non-sample materials into the path of the beamline. Surface acoustic wave techniques have also been shown to be useful to present room temperature MX samples to the X-ray beam at both synchrotron and XFEL sources and have been shown to be non-destructive in respect to protein crystals^14,15^.

A technique which does not introduce any crystalline non-sample material into the beamline is acoustic levitation, where the sample is presented without contact from external supports as has been previously demonstrated for MX at the Swiss Light Source^16^. This builds on other X-Ray scattering experiments with levitated samples such as at the MAX II, Sweden^17^ and BESSY, Germany^18,19^. It has also been used for small-molecule X-ray diffraction experiments (for example Klimakow^20^ and Nguyen^21^). Such approaches however, have not found widespread adoption owing to the fact that they typically require the construction of two frequency matched Langevin horns, a costly and challenging process. Furthermore, as the Langevin horns’ frequency shifts with temperature by ≈4 Hz/C^22^ a pre-experiment stabilisation time and a controlled temperature and humidity environment are required. Changes in temperature also impact the stability of the standing wave nodes due to the corresponding change in the speed of sound^23^. Langevin horn systems also impart significant energy into the entrapped fluids yielding high or uncontrolled temperatures during experiments.

A new generation of low cost, low power, portable and self-contained acoustic levitation devices is demonstrating renewed opportunities for the approach. The TinyLev system^24^ offers contact-free manipulation with no pre-experiment conditioning.

Whilst acoustic levitators are capable of supporting almost any liquid in a suitably sized droplet, delivering such a droplet to a system can be challenging. This is particularly true when also trying to incorporate a protein crystal inside the delivered droplet. Protein crystals are typically grown in solvents with high surface tensions and therefore, the crystal solution often remains attached to the pipette tip during loading into the levitation field. Droplet stability has been shown to be improved by adding a coating of oil^25^ which also brings the potential benefit of a significant reduction in sample evaporation rates (as demonstrated for octadecanol^26^).

In this study we demonstrate an application of the acoustic trap system as described by Marzo^24^. Protein crystals are suspended in single, microlitre sized droplets, coated in silicone oil and presented acoustically to the X-ray beam. Two sample forms were investigated: small numbers of 100 to 800 μm crystals and also a high density slurry of 10 to 15 μm crystals. We have found that the incorporation of silicon oil coat around the protein crystal solution dramatically increases the ease of delivering the levitating drop incorporating the sample crystal. This method has solved a significant barrier to entry for acoustically levitating MX samples and will open up new avenues of automated sample delivery. The coating will allow for the universal presentation of liquid samples regardless of their surface tension. The device both suspends the sample and also imparts a modest but sufficient motion to allow for a complete, high-quality, rotation style dataset to be recorded and processed in an efficient and routine manner. We have also determined the optimum system voltage to trap relevant sample volumes to maximise the applicability of the encapsulated droplet approach.

## Results

Results of each of the experiments are presented in the following sections.

### Optimisation of levitator voltage

**Figure 1:**
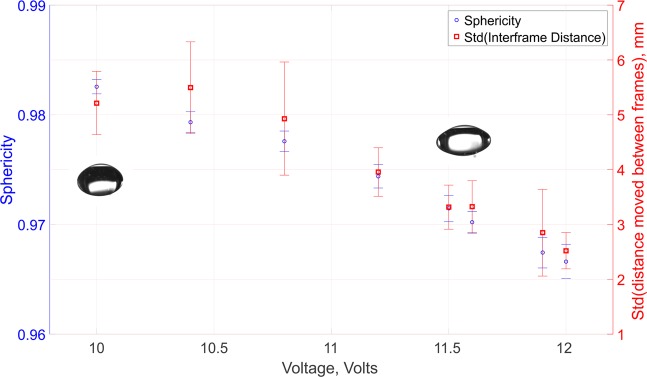
Plot of the droplet sphericity and stability as a function of applied voltage. Voltages above 11.5 V provide the greatest stability but with an ever increasing effect on the sphericity. This voltage was used for all synchrotron experiments.

It is well observed that, in addition to numerous system parameters, the transducer power has a significant effect on the shape and stability of droplets within acoustic levitation systems. This is due to the changes in the resulting sound pressure levels, which was previously explored using the Langevin systems^27,28^. The same was true of the TinyLev system, and the data in Fig. 1 shows the relationship between droplet sphericity (as determined using Equation 1) and spatial stability, as a function of the applied voltage for a levitated silicone oil coated water droplet. In this work, 350 cSt silicone oil was used as it offered the optimum compromise (from a range of silicone oils varying from 10 cSt to 10,000 cSt) for delivering a sufficient thickness of coating to the droplet whilst not requiring extensive time to pipette. It may be favourable in other MX experiments utilising this method to use alternative oils to ensure compatibility with the elements of the crystallisation solution or to further reduce background signals in exchange for less favourable reduction in evaporation rates.

Throughout the droplet tracking experiments, no measurable evaporation of the samples was seen, suggesting that the coating of silicone oil was sufficient to limit sample loss to the environment. An off line experiment monitoring the evaporation of water and ethanol droplets with and without silicone oil coating is presented in ESI1 confirming these findings; showing less than 5% change over the 50 minutes which an uncoated droplet took to evaporate until unconfined. The use of non-hygroscopic oils should theoretically eliminate evaporative processes entirely.

It was seen that there was a decline in the droplet sphericity as a function of applied voltage which remained above 97% until 11.5 V. The stability however, improved twofold up to 11.5 V and then fell within error estimates up to 12 V. This suggested that 11.5 V represented the best compromise between maintaining drop stability and sphericity for these samples, and was therefore used for all synchrotron experiments. This results in the presentation of an oblate cross section to the beamline of (2.30 ± 0.01) mm by (1.50 ± 0.02) mm. It is however likely, that this voltage will not be optimum for samples which have radically different densities or surface tensions such as crystallisation solutions with high concentrations of volatile components or high molecular weight poly-ethylene glycols.

Minor imperfections in the efficiency of the 72 ultrasonic transducers, slight irregularities in sample density, morphology and local air turbulance also tend to impart a slight rotation of the droplet. This rotation was exploited during X-ray data collection to allow the collection of a complete X-ray diffraction dataset (discussed in the following section). The suspension of such droplets against gravity using this system requires little energy and thus imparts no significant heating to the sample (confirmed by non-recorded thermal imaging), allowing for true ambient investigation of the protein structure.

### Macromolecular crystallography

**Figure 2:**
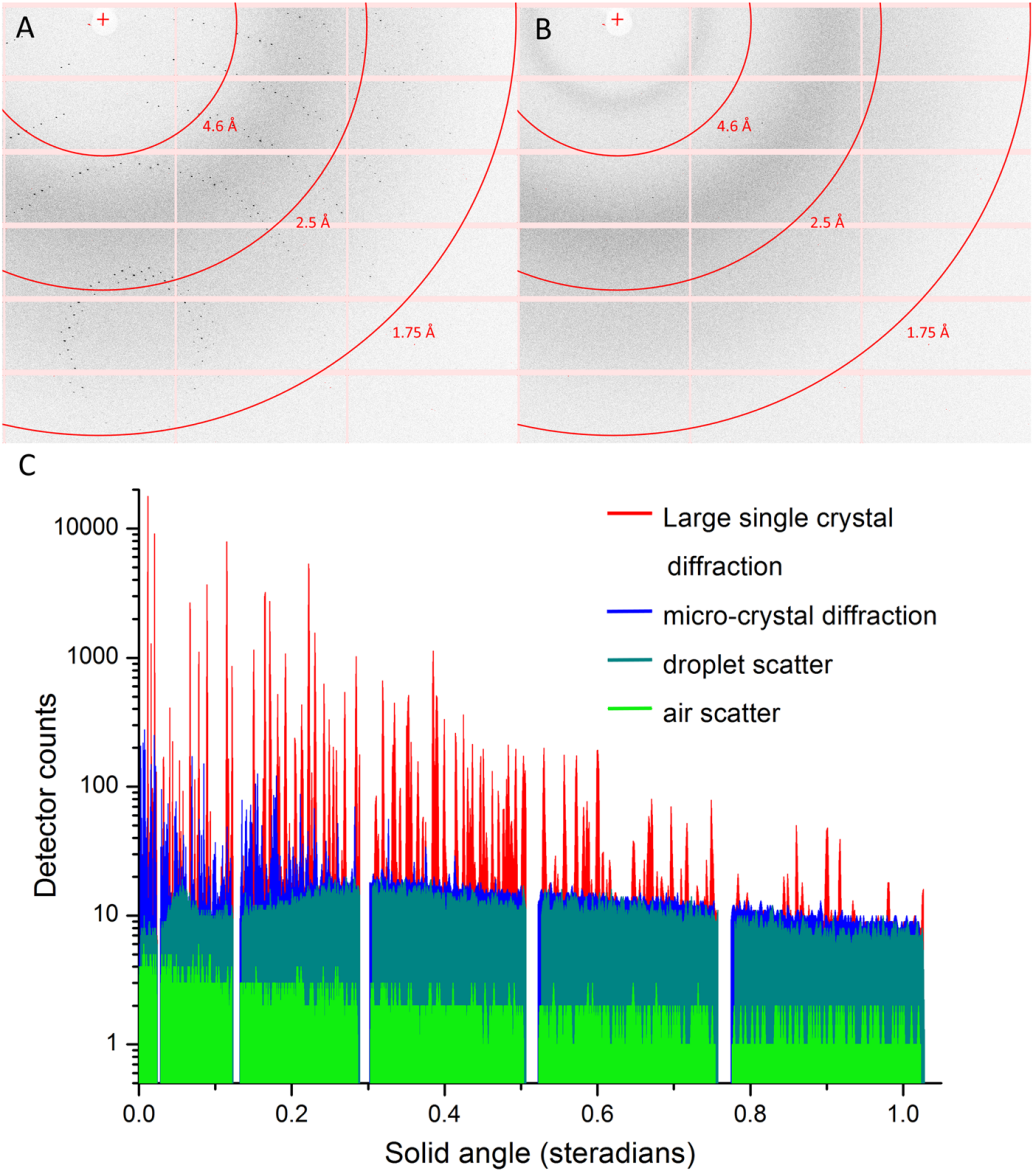
Composite figure illustrating sample diffraction and background scatter. (**A**) Section of detector image showing example large crystal diffraction used for structure deposition. (**B**) Section of detector image showing example micro-crystal diffraction. (**C**) X-ray scatter from levitating drop experiments as image sections taken downwards from beam-centre to edge of detector and plotted as a solid angle. The diffraction profiles represent maximum pixel values recorded over the structure deposition dataset (red) and the micro-crystal slurry dataset (blue). For comparison, 100 image averaged scatter profiles from a drop of crystal buffer with silicone oil preparation (grey) and air scatter (green) are also shown.

The lysozyme crystal structure (structure factors and coordinates have been deposited under Protein Data Bank (PDB) entry 6QQ3^29^) was determined from a single continuous collection run of 5,000 images (yielding 4,086 merged and scaled diffraction patterns) on a drop containing an estimated 4–6 crystals with dimensions ranging from 100 to 800 μm. The statistics are presented in Table 1. An example of the diffraction recorded on the detector is shown in Fig. 2(A). We have exploited serial crystallography data analysis tools to analyse detector frames on an individual basis, given the absence of a fixed axis of rotation for the levitating droplet. Although all patterns from the best single data collection run were included to optimise the data metrics of the deposited dataset, as few as 500 images were sufficient for a 96.7% complete dataset to 1.69 Å. This finding highlights the potential for the method to record structural data in an extremely rapid and efficient manner, particularly if a continuous rotation of the drop occurs. Future iterations of the system aim to eliminate this uncontrolled rotation and instead impart an induced, constant rotation by modifying the design of the transducer array, relative transducer phasing and drive electronics to further facilitate this process. ESI2 provides a movie of a droplet spinning outside of the synchrotron setup but with otherwise identical parameters to demonstrate the motion experienced.

**Table 1:** Summary statistics for diffraction data collection, processing and refinement.

Data collection	
Beamline	BLI24 (Diamond Light Source)
Wavelength (Å)	0.9686
Incident flux (photons per s)	3 × 10^11^
Beam size (m)	50 × 50
Exposure time (ms)	10
Detector	Pilatus3 6 M
Sample-detector distance (mm)	325
No. frames collected	5,000
No. integrated (merged) frames	4,096 (4,086)
**Scaling and merging**	
Space Group	P4_3_2_1_2
Unit cell parameters (Å)	79.4, 79.4, 37.9
Resolution range (Å)	39.74–1.53 (1.56–1.53)
R_split_	0.101 (0.549)
CC_1/2_	0.982 (0.665)
(I/*σ*(I))	3.52 (0.71)
Multiplicity	101.5 (8.84)
Completeness (%)	99.5 (95.0)
**Refinement**	
No. reflections	18,767
No. non-H atoms (protein)	2,480
No. non-H atoms (water)	76
R/R_free_	0.179/0.203
R.m.s.d., bond length (Å)	0.005
R.m.s.d., bond angles ()	0.767
Ramachrandran outliers (%)	0
Side chain outliers (%)	0.8
PDB code	6QQ3

**Figure 3:**
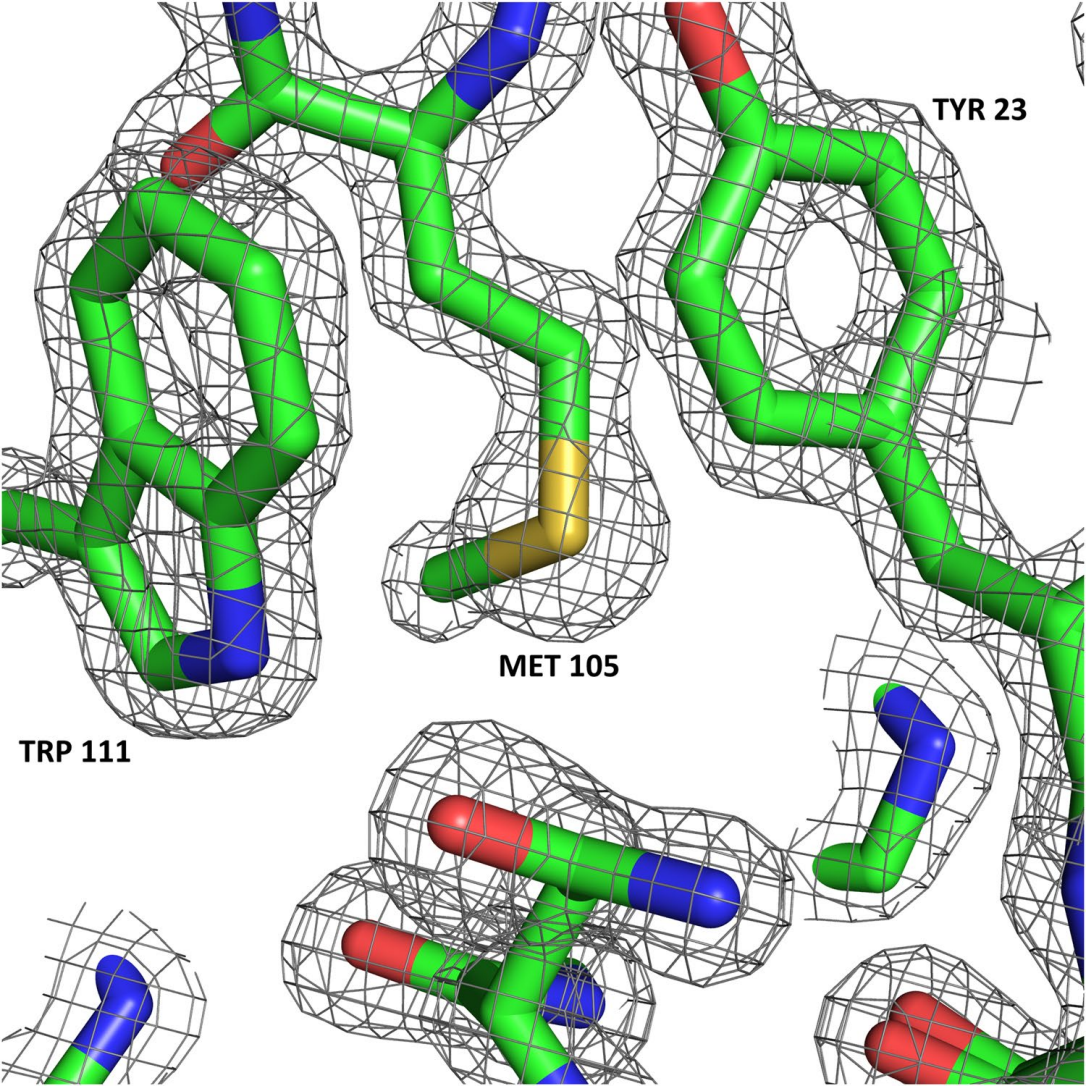
Example electron density (2Fo-Fc map contoured at 2.0 *σ*) obtained from an acoustically levitated lysozyme crystal in an oil coated droplet of mother liquor. PDB ID: 6QQ3^29^. Graphics produced using PyMOL^42^.

Example electron density from the structure (available as PDB entry 6QQ3^29^) is shown in Fig. 3. This demonstrates the device’s ability to produce high quality structural information from acoustically supported microlitre volumes in a completely non-contact manner at room temperature.

**Figure 4:**
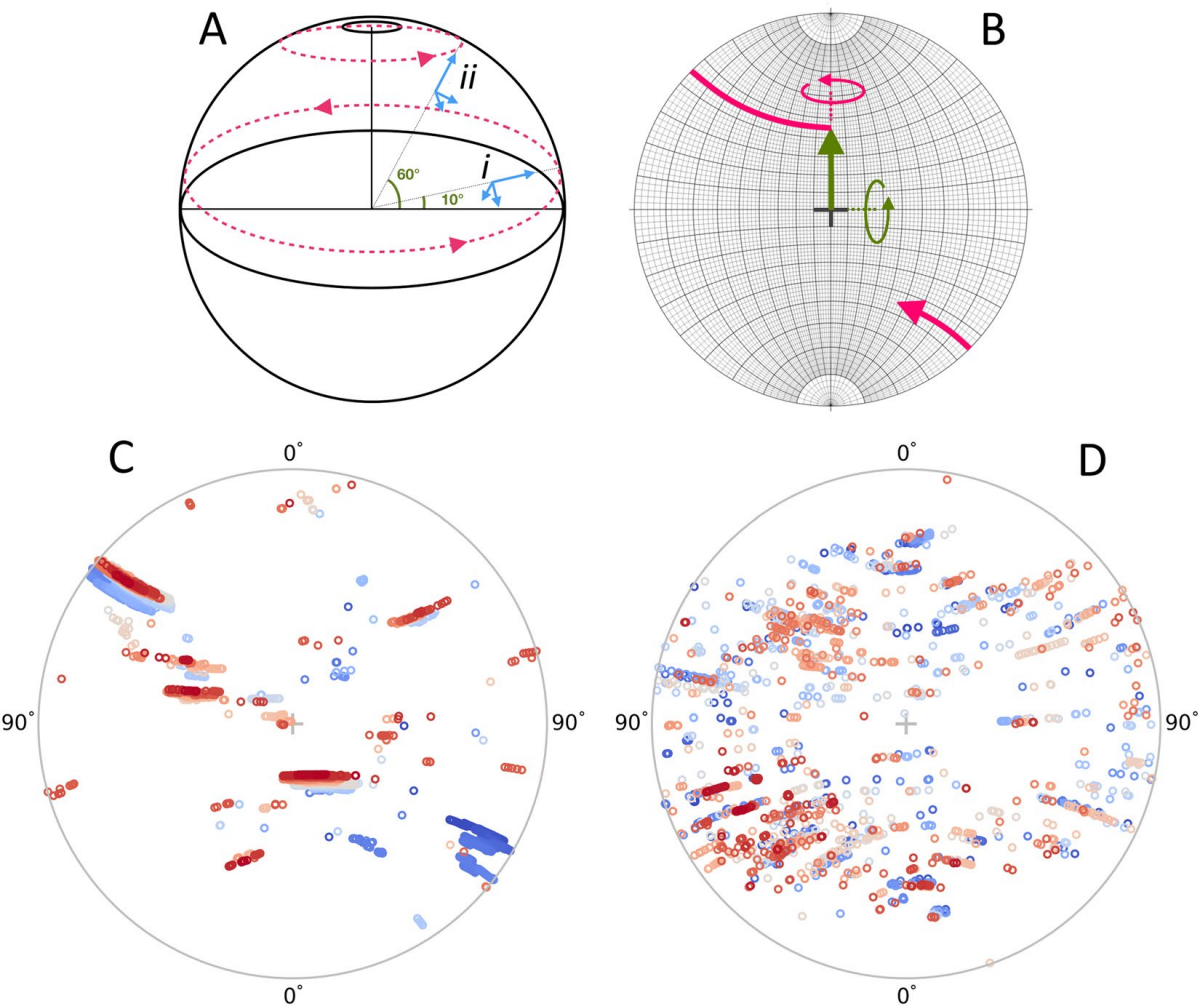
Stereographic projections of crystal orientations and motions. (**A**) Schematic showing two crystals *i* and *ii* inside a spherical drop angled 10º and 60º respectively to the horizontal. As the crystals rotate about a vertical axis, projections from them describe circular paths on the surface of the sphere. (**B**) A Wulff net^43^ where the surface of a hemisphere of A has been projected onto the page, such that lines of longitude describe rotations about a horizontal axis (Green) and lines of latitude trace rotations about a vertical axis (pink). The [001] *hkl* reflection of the crystal is used as the reference. If the reflection is initially aligned to the beam at the origin (marked by central cross) then a rotation of 45º of this reflection about the horizontal axis will track the path of the large green arrow. From this direction the large pink arrow describes a further rotation about the vertical axis of 135º and as the reflection moves through 90\deg it appears on the lower half via its backward projection. On this plot rotations about the beam would track circles concentric to the cross. (**C**): Stereographic projection showing the direction of the [001]* hkl* (indexed in P1) from the 5,000 image collection used for the structure deposition, consisting of 4096 diffraction patterns. (**D**) Similar plot but from a 10,000 image collection run on a slurry of micro-crystals consisting of 1498 diffraction patterns. Both plots suggest rotations about the vertical axis as the patterns of points fit circles of latitude. (C) is dominated by three clusters, the largest of which (straddling both hemispheres can be assigned to 2260 patterns. (D) shows many more different tracks of points implying many more different lattices contributing to the dataset. Colours represent the recorded sequence of images: blue through pink to red. Plots (C) and (D) produced using the dials.stereographic_projection module^34^.

Processing *via* a serial method assumes each detector frame to be an individual experiment and refines an independent crystal lattice orientation for each instance. A more detailed analysis of the 5,000 image structure solution run reveals the presence of multiple lattices and their respective motions during data collection. Figure 4(C) shows a stereographic projection that plots the direction of the [001] *hkl* of each integrated lattice as indexed in P1, so as not to show symmetrically related reflections. The clusters on the plot suggest the presence of multiple crystals but could also represent crystals leaving the beam and then re-entering at a different orientation. On any one image a maximum of three lattices are detectable, occurring on 154 images and indicating an absolute minimum of 3 crystals in the drop. Assessing the number of crystals visually was not possible so the success of the transfer step from the crystallisation tray was uncertain. The largest continuous run from a single lattice is 2,260 frames (maximum separation of 3 between consecutive images). An animation has been constructed from these data to illustrate the motion of this crystal and is included in ESI3. Across the entire collection run and accounting for discontinuities, the mean oscillation step between frames is 0.64 (s.d. 0.59) or 64/s with a maximum oscillation step of around 2 or 200/s.

The 2,260 frames of single lattice data allows us to estimate a dose on this crystal using the parameters reported in Table 1, the RADDOSE-3D program^30^ and estimates of the crystal sizes: a crystal of 100 × 100 × 200 μm gives a diffraction weighted dose of 210 kGy and a crystal of 200 × 400 × 800 μm gives a diffraction weighted dose of 150 kGy. This assumes the crystal remains centred on the beam as it rotates, any misset, which seems quite likely in this case, would bring additional sample volume to the beam. Exploiting the fact that near complete data could be obtained by relatively few images, further investigation of radiation induced changes to the protein structure was undertaken by comparing datasets formed from the first and last 750 images of the 2,260 image run. Isomorphous difference maps showed no significant or obvious features. However, a comparison of the two 750 image subsets did reveal a drop with an I/*σ*I from 6.06 to 4.23 over a resolution range of 39.75–1.70 Å and from 1.14 to 0.69 in the highest resolution shell (1.73–1.70 Å). This drop in I/*σ*I suggests that although the obvious effects of radiation damage did not appear to manifest in the electron density, they were still present.

The potential of the device with micro-crystals was also explored with a data collection on a micro-crystal slurry of 10–15 m lysozyme crystals. Raster scanning the droplet through the beam revealed the micro-crystals sedimenting and diffraction from the bottom was powdery and not readily interpretable. However, by positioning a 20 × 20 μm beam just above the sedimented region, individual lattices could be recorded, indexed and integrated with a moderate hit-rate. An example of diffraction recorded on the detector is shown in Fig. 2(B) with individual diffraction spots not easily visible, in contrast to the large crystal in Fig. 2(A). In total, 1,498 useful patterns were obtained from a 10,000 image collection from a single drop; enough for a complete dataset to a resolution of approximately 2.6 Å. Figure 4(D) shows the individual lattice orientations and in contrast with the deposition dataset (Fig. 4(C)), a large number of different crystals are suggested; each contributing a smaller proportion of the total data. The limited resolution seen from the micro-crystals is a function of the significant background scatter from the liquid volume of the drop and this is illustrated in Fig. 2(C); a comparison of the scattering from the deposition data, the micro-crystal data, an oil-encapsulated droplet of buffer and air scatter. The background scatter from the drop is about 6 times larger than that of an air path, and whilst the large crystal diffraction is seen to extend beyond the edge of the detector, the much weaker diffraction from the micro-crystals disappears into the droplet-scatter at much reduced angles.

## Discussion

We have presented results demonstrating the potential of acoustically levitated, oil encapsulated drops as a physical mount free method for Macromolecular Crystallography experiments. Levitation can enable efficient room temperature *in situ* X-ray data collection, in part by exploiting the fact the sample motion is not about a fixed single axis, thus potentially accelerating the acquisition of a complete set of crystal reflections.

The oil-encapsulation approach neatly side-steps the issue of droplet surface tension that can adversely affect device loading and sample stability. Additionally the non-contact nature of the technique offers advantages to traditional presentation methods utilising cryogenic sample fixed on pins^31,32^ or on physical films^33^. Furthermore, oil encapsulation of droplets significantly lowers evaporation rates enabling data collection on volatile solutions and removes the complication of dehydration and variable sample volume. Similarly, the minimal energy which is imparted into the droplet in acoustic suspension ensures that there is little droplet heating, greatly reducing the risk of sample damage that can come with higher power Langevin horn systems and improving the relevance of the resulting data. The light-weight and low-volume of the TinyLev device enabled easy location of the levitating drop to the X-ray beam since the existing beamline sample positioning stages could be used. Indeed, even raster scanning was possible to quickly assess variation in density of sample over the droplet cross-section.

Although serial methods were used for data analysis, the final dataset was more readily derived by virtue of the crystal motion. This motion allows for a larger slice of reciprocal space to be recorded than would be from a static sample with a monochromatic X-ray beam. As a result the mean oscillation width observed here of 0.64 enabled the collection of complete data with hundreds of images rather than the thousands typically required for structure determination with serial stills. With dose estimates in the hundreds of kGy range for the deposition dataset, some radiation induced changes would be expected at room temperature and a drop in I/*σ*I was observed here. However, these estimates are compromised by not being able to visualise the diffracting crystal and the crystal not being aligned precisely to the beam. Currently, integrating diffraction data with an oscillation model when the crystal is rotating about a variable axis and with varying direction and speed represents a non-trivial analysis problem. Developments within the open source Diffraction Integration for Advanced Light Sources (*DIALS*)^34^ software framework are being explored to improve the ease of such analysis.

The restricted resolution seen in the micro-crystal diffraction indicates that the drop volume and its contribution to background scatter is currently a limitation. We anticipate being able to reduce the droplet volume (and concomitantly the useful crystal volume) with theoretical estimates suggesting minimum droplet sizes in picolitres. This will create opportunities for studies of the more dynamic processes accessible in ambient conditions, such as *in crystallo* enzyme-substrate turnover experiments, which are greatly dependent on diffusion rates. Additionally, to enhance the applicability of the method, future work will explore automated delivery of droplets to the acoustic nodes. Automated device loading would significantly increase throughput, potentially moving this technique towards serial injection methods but with the huge advantage of being able to hold samples at the point of X-ray interaction. A final enhancement will be a more effective control of the droplet motion to optimise diffraction data acquisition, with the aim of continuous rotation in one direction at a constant speed appropriate for the detector readout rate.

We believe the system to be suitable for deployment on other high intensity X-ray sources operating in ambient conditions, owing to its compact, fully self-contained nature, minimal power delivery and has potential to become a readily adopted sample presentation system for Macromolecular Crystallography experiments.

## Equipment Setup

In this section, the sample presentation equipment is described and its effect on the fluid droplets analyzed.

### Levitation system

**Figure 5:**
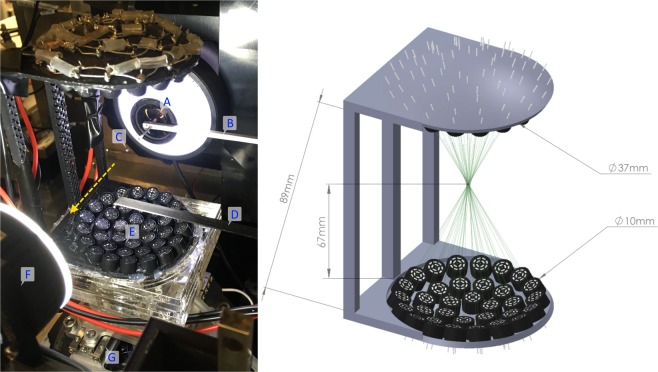
Left: Photograph showing the TinyLev system mounted on the I24 beamline with the X-ray beam path marked with a yellow dashed arrow. Components as labelled: (**A**) High-magnification viewing system, (**B**) X-ray scatter-guard, (**C**) levitating drop, (**D**) beamstop (out of position), (**E**) TinyLev Transducer array, (**F**) backlight (retracted during data collection), (**G**) sample positioning stage. Right: Model of the acoustic levitation system (**E**) used in this work annotated with key dimensions and showing the focal point of the transducer array.

A full description of the acoustic levitation system utilized in these experiments is described by Marzo *et al*.^24^ to which the reader is directed for construction detail. For application to the beamline, an acrylic mounting system is produced allowing the device to be attached to the existing sample positioning stages (capable of positioning attached samples or devices at different angles, always in the horizontal plane, at variable vertical positions). The electronics are mounted away from the system to minimize the equipment near the X-ray beam. A photograph of the system as mounted at the beamline is shown in Fig. 5.

### Droplet confinement voltage

The system has a variable input voltage which has a direct influence on the acoustic pressure imparted on trapped samples. By changing the voltage it is possible to confine fluids of different densities and size. This however, results in a change to the droplet shape tending from a spheroid to an oblate morphology.

The optimum voltage needed to acoustically trap droplets is a trade off between applying sufficient voltage to overcome gravitational effects on the droplet and reach relative spatial stability whilst maintaining as spherical a droplet shape as possible, such that the sample crystal is readily found at the lowest point. We determined the optimum voltage for this experiment by capturing several series of images of the droplet confined by an acoustic field generated by different input voltages. A camera (DCC1645C, Thorlabs, USA) is focused onto the central acoustic trap and images collected as a multi-page Tiff file. These files are imported into MATLAB (Mathworks, USA) where the blue channel (as this gives the greatest droplet contrast) is thresholded and the resulting image closed (with a 50 pixel diameter disk operation) before being skeletonised for ellipse fitting using the fit ellipse function^35^. The outputs of this function are then used to calculate the sphericity according to Equation 1 and to approximate the spatial stability of the droplet by taking the standard deviation of the change in centre point between frames. ESI4 provides a movie comparing two droplet voltages and showing half original droplet image and half the output of the fit to demonstrate the suitability of this method.

There is a trade off between higher voltages which ensure stable entrapment (up to the point at which the droplets are split into smaller volumes) at the expense of maintaining sphericity and providing sufficient acoustic pressure to levitate their mass. We present a combined plot of sphericity and stability for a silicone oil coated water droplet to determine the optimum range of voltages suitable for such an experiment.

1 $${\rm{\Psi }}=\frac{2\sqrt[3]{a{b}^{2}}}{a+\frac{{b}^{2}}{\sqrt{{a}^{2}-{b}^{2}}}ln(\frac{a+\sqrt{{a}^{2}-{b}^{2}}}{b})},$$

where a and b are the semi-major and semi-minor axes respectively as determined from the ellipse fitting function.

## Methods

The experimental method employed to prepare a suitable crystal containing droplet and present it to the synchrotron beam is described below.

### Sample preparation

Commercial lysozyme from chicken egg white (CAS Number 12650-88-3, Sigma-Aldrich, UK) was initially resuspended to a concentration of 25 mg · mL^−1^ in 100 mM Na Acetate pH 3.0. Large lysozyme crystals (100–800 μm longest dimension) were grown using seeding. Micro-crystals were initially grown by mixing the protein solution 1:1 with 28% (w/v) NaCl, 8% (w/v) PEG 6,000, 100 mM Na Acetate pH 3.0 in a centrifuge tube. After 1 hour the resultant highly concentrated microcrystalline slurry (longest dimension <5 μm) was diluted 1 × 10^7^ fold. This seed solution was then mixed with 10% (w/v) NaCl, 25% (w/v) ethylene glycol, 100 mM Na Acetate pH 4.8 and with protein solution (75 mg · mL^−1^ in 100 mM Na Acetate pH 3.0) in a ratio of 1:2:3 μL (seed:precipitant:protein). The drops were then incubated overnight at 18 C and harvested the following day. The 10–15 μm crystalline slurry was prepared by mixing lysozyme solution (25 mg · mL^−1^ in 100 mM Na Acetate pH 3.0) with precipitant (16.8% (w/v) NaCl, 4.8% (w/v) PEG 6,000, 60 mM Na Acetate pH 3.0), 1:1 in a centrifuge tube. Crystals appeared after 1 hour and were used the following day.

In order to impart the silicone oil coating described in the levitation section to the droplet, 10 μL pipette tips were coated with a commercial chemical hydrophobising agent (Rain-X, Illinois Tool Works, USA) which was allowed to dry and prevented the adhesion of water based droplets (and thus also the extraction of the aqueous core from the silicone oil layer) to the tip. 2.5 μL of 350 cSt Silicone Oil (Sigma Aldrich, UK) was then pipetted and discarded and a thin layer retained by the tip. Gravimetric analysis suggests that this leaves approximately 0.35 μL of silicone oil coating the internal tip surface. 2.5 μL of the sitting drop or crystalline slurry was then collected using the same tip and the coated droplet transferred to the central acoustic trap which was estimated to consist of 4% silicone oil. The levitating droplet was then aligned with the beam using the beamline’s sample positioning stages on to which the TinyLev device had been attached with a 3D printed adaptor mount.

### Synchrotron data collection

All MX experiments conducted for this work were performed on I24 at Diamond Light Source, Harwell, UK; a tunable microfocus synchrotron beamline. The incident area of the 0.9686 Å X-Ray beam was set to 50 × 50 μm (full-width-half-maximum) focused using a pair of Kirkpatrick-Baez mirrors, a scatterguard 5 (Left, [B]) serves to clean rather than shape the beam profile. Diffraction data were collected using a Pilatus3 6 M detector running at 100 Hz using all 5 × 12 detector modules. Full MX experiment parameters are shown in Table 1. Temperature and relative humidity at the sample position in the beamline hutch were recorded at 21.4 C and 30% respectively, at the start of the experiment and later found to vary with a standard deviation of +/−0.2 C and +/−3% over a 24 hour period.

Initially raster scans were performed over the cross section of droplets to determine the location of crystals and it was found that despite the rotational motion, crystals sedimented under gravity towards the bottom of the droplet. This area was then used as the target for a data collection run on a newly mounted droplet containing an estimated 4–6 crystals. 5,000 diffraction images were collected in 50 seconds with the detector operating in a free-running, shutterless manner. During this time no physical change was observed visually in the droplet, as observed on the integrated on-beam-axis high magnification camera system. The droplets were stable over time at ambient temperature with no requirement for humidity control. The silcone oil coating did not measurably increase background X-ray scatter, the dominant factor being the path length through the droplet.

### Diffraction data processing

The images containing the diffraction data were analysed with the open source Diffraction Integration for Advanced Light Sources (*DIALS*)^34^ software package using dials.stills_process to perform diffraction spot finding, space group and unit cell indexing, determination of the crystal rotation matrix, and reflection integration as proposed by Brewster *et al*.^36^. Individual integration files were merged and put on a common scale using the program PRIME^37^. Example diffraction can be seen in Fig. 2.

### Structure solution

The crystal structure was solved using molecular replacement with PDB entry 5KXO^38^ truncated to polyalanine. Model building was completed using phenix.autobuild^39^ and Coot^40^ with refinement performed with phenix.refine^41^. Statistics for data collection and refinement are presented in Table 1.

## Supplementary information

ESI1

ESI2

ESI3

ESI4
