# Supplementary material for: Non-Contact Universal Sample Presentation for Room Temperature Macromolecular Crystallography Using Acoustic Levitation
Source: Sci Rep. 2019 Aug 27;9:12431. doi: 10.1038/s41598-019-48612-4 (PMC6712007; doi:10.1038/s41598-019-48612-4)

Non-Contact Universal Sample Presentation for Room Temperature Macromolecular Crystallography Using Acoustic Levitation

R. H. Morris[1,*], E. R. Dye[1], D. Axford[2], M. I. Newton[1], J. H. Beale[2], and P. T. Docker[2]

[1]School of Science and Technology, Nottingham Trent University, Nottingham, NG11 8NS, UK.
[2]Diamond Light Source, Harwell Science and Innovation Campus, Oxfordshire, OX11 0DE, UK
*rob.morris@ntu.ac.uk

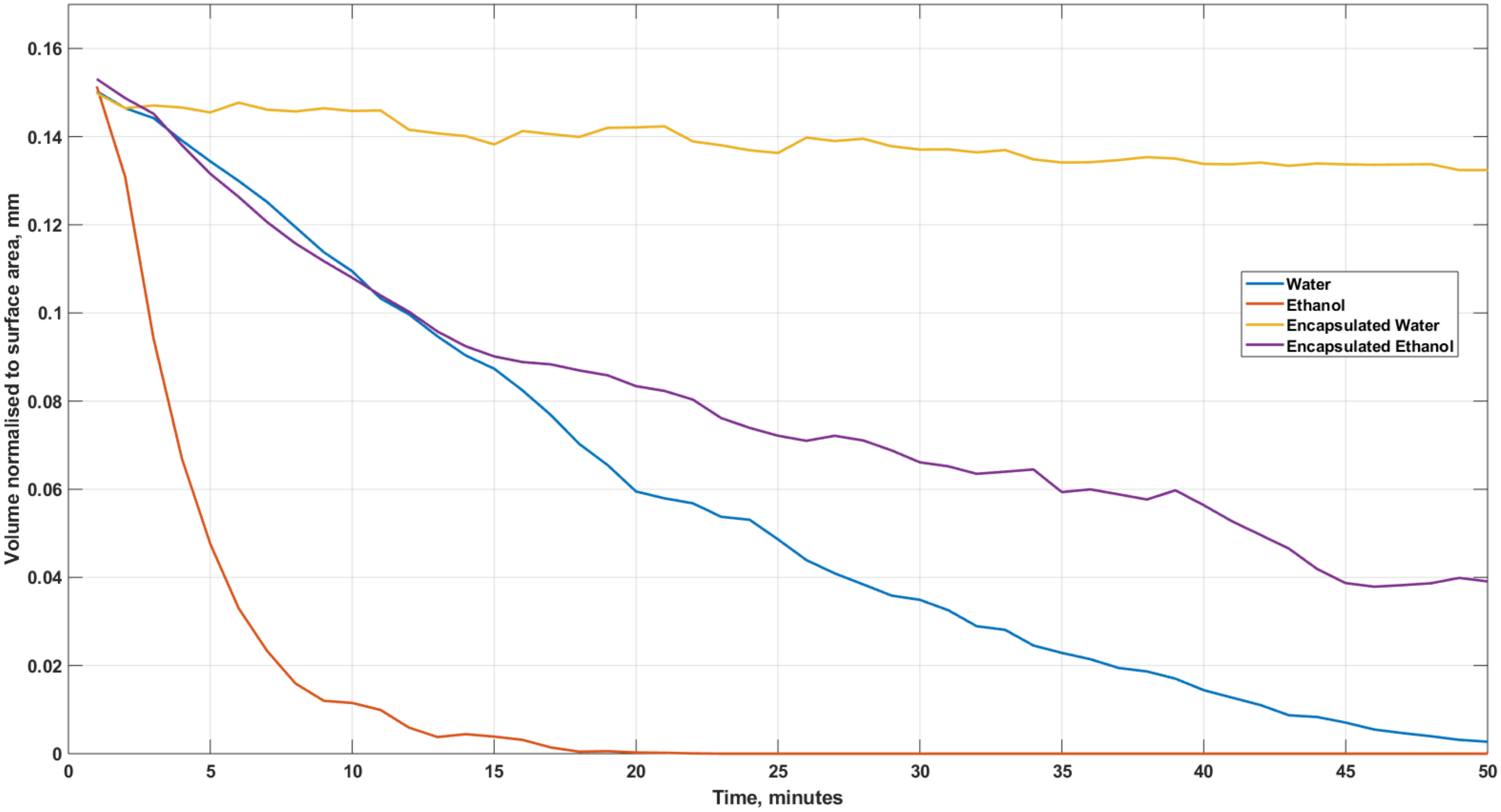

Supplement: Supplementary file 1 — ESI1 [file 41598_2019_48612_MOESM1_ESM.pdf]
